# Defining the optimal method for measuring baseline metabolic tumour volume in diffuse large B cell lymphoma

**DOI:** 10.1007/s00259-018-3953-z

**Published:** 2018-02-19

**Authors:** Hajira Ilyas, N. George Mikhaeel, Joel T. Dunn, Fareen Rahman, Henrik Møller, Daniel Smith, Sally F. Barrington

**Affiliations:** 1 Department of Nuclear Medicine, Guy’s and St Thomas’ NHS Foundation Trust, London, UK; 2 Department of Clinical Oncology, Guy’s and St Thomas’ NHS Foundation Trust, London, UK; 3 Kings College London and Guy’s and St Thomas’ PET Centre, School of Biomedical Engineering and Imaging Sciences, King’s College London, King’s Health Partners, London, UK; 4 Department of Cancer Epidemiology and Population Health, King’s College London, King’s Health Partners, London, UK

**Keywords:** Positron-emission tomography, Lymphoma, Diagnosis, Imaging

## Abstract

**Purpose:**

Metabolic tumour volume (MTV) is a promising prognostic indicator in diffuse large B cell lymphoma (DLBCL). Optimal thresholds to divide patients into ‘low’ versus ‘high’ MTV groups depend on clinical characteristics and the measurement method. The aim of this study was to compare in consecutive unselected patients with DLBCL, different software algorithms and published methods of MTV measurement using FDG PET.

**Method:**

Pretreatment MTV was measured on 147 patients treated at Guy's and St Thomas’ Hospital. We compared 3 methods: SUV ≥2.5, SUV ≥41% of maximum SUV and SUV ≥ mean liver uptake (PERCIST) and compared 2 software programs for measuring SUV ≥2.5; in-house ‘PETTRA’ software and Hermes commercial software.

**Results:**

There was strong correlation between MTV using the 4 methods, although derived thresholds were very different for the 41% method. Optimal cut-offs for predicting PFS ranged from 166–400cm^3^. All methods predicted survival with similar accuracy. 5y-PFS was 83–87% vs. 42–44% and 5y-OS was 85–89% vs. 55–58% for the low- and high-MTV groups, respectively. Interobserver variation in 50 patients showed excellent agreement, though variation was lowest using the SUV ≥ 2.5 method. The 41% method was the most complex and took the longest time.

**Conclusion:**

All methods predicted PFS and OS with similar accuracy, but the derived cut-off separating good from poor prognosis varied markedly depending on the method. The choice of the optimal method should rely primarily on prognostic value, but for clinical use needs to take account of ease of use and reproducibility. In this study, all methods predicted prognosis, but SUV ≥ 2.5 had the best inter-observer agreement and was easiest to apply.

## Introduction

Diffuse large B cell lymphoma (DLBCL) is the commonest subtype of lymphoma, representing 30% of lymphoid malignancies [1]. There has been a significant improvement in cure rates in recent years, with the addition of rituximab to cyclophosphamide, adriamycin, vincristine, and prednisone (CHOP) chemotherapy. However, a significant proportion of patients will progress or relapse after R-CHOP [2, 3] and long-term cure rates are only about 60% [4]. Whilst first line treatment has become more successful, salvage therapy after up-front rituximab has become less effective [5, 6]. It is important therefore to be able to reliably assess both pretreatment risk and identify patients at high risk of progression or relapse early to tailor treatment and test alternative approaches [7].

The International Prognostic Index (IPI) is currently used for estimating pretreatment risk, despite the fact that IPI often does not reliably predict individual patient outcome because DLBCL tends to behave heterogeneously [8]. Other factors that can predict prognosis, such as cell of origin or specific translocations, e.g. double-hit lymphoma (*myc* and *bcl-2* translocations), have been identified but have not resulted in therapeutic advances as yet [9, 10].

The response to treatment in DLBCL has great prognostic value. Complete remission at the end of chemotherapy is associated with a high rate of progression-free survival (PFS) [11], but this information is obtained too late for choosing treatment. Positron emission tomography (PET) has been found to be useful in early monitoring of treatment for aggressive lymphomas [12]. In Hodgkin lymphoma, published multicentre trials support the use of early ‘interim’ PET for response-adapted treatment [13, 14]. However, in DLBCL, whilst initial reports suggested interim PET could reliably predict chemoresistance to CHOP [15, 16], later reports suggested the introduction of rituximab might affect the interpretation of “positive” interim PET scans [1, 17, 18]. Currently, the PFS of patients with a positive interim scan treated with R-CHOP is around 50% at 2–5 years [17, 18]. Attempts to standardise PET reporting [11, 19] and improve the positive predictive value of interim PET using semi-quantitative approaches [20] have not been sufficiently improved to enable interim PET to discriminate a group with poor prognosis in whom a change of treatment would be warranted [21, 22].

Baseline imaging characteristics can also predict outcome [23], including tumour burden [11]. The MInT study demonstrated a linear relationship between maximum tumour dimension and prognosis in patients treated with R-CHOP [4]. More recently metabolic tumour volume (MTV) has been identified as a promising baseline prognostic factor [11, 24, 25] that is superior to size-defined bulk [26, 27]. The high contrast afforded by 18F fluorodeoxyglucose (FDG) PET imaging may overcome some of the interobserver variability reported when segmenting tumour regions using computed tomography (CT) and it appears that PET is closer to the ‘ground truth’ when a tumour is delineated using PET compared to CT in solid tumours [28, 29]. The use of PET automatic delineation methods may also reduce interobserver variability [30].

Several methods have been proposed to measure MTV and applied in selected patients with large cell lymphoma. This has resulted in different cut-offs for MTV that separate good from poor prognostic groups [24–26]. We recently reported our experience measuring MTV using software developed in-house. We combined baseline MTV with early response assessment using Deauville criteria in consecutive unselected patients with DLBCL treated with R-CHOP at a single institution [26] using quality assurance methods developed for clinical trials [31]. Using this approach, a third of patients were found to have high baseline MTV with incomplete early metabolic response after 2 cycles of R-CHOP and 5y-PFS of only 30% [26].

Validation of these data will require large patient numbers and involvement of international groups. Standardisation of the methodology for MTV is crucial for this endeavour, as previously occurred with the assessment of PET response using the Deauville criteria [11, 19]. Methods also need to be available using commercial software and be robust and easy to use in daily practice.

The aim of this study therefore was to:

1. Compare the reproducibility of measuring total MTV using in-house software (as previously reported) [26] and commercially developed software (Hermes Medical Solutions, Sweden)
2. Compare various published ways to perform MTV segmentation
3. Assess inter-observer variability in MTV measurement and ease of use of different methods
4. Compare accuracy of the various MTV segmentation methods to predict PFS and overall survival (OS) in DLBCL [25, 26, 32, 33]

## Patients and methods

Consecutive patients with DLBCL treated with R-CHOP at Guy’s and St Thomas’ NHS Trust from 2005 to 2012 were included [26]. Baseline PET/CT scans were acquired after a 6-h fast and 90 min after administration of FDG produced in an on-site cyclotron.

Images were acquired from the base of the skull to upper thighs using DST or VCT scanners (General Electric, Waukesha, WI, USA) for 5 minutes per bed position with separate head and neck views, if required. CT parameters were 140 kV; 115 mA; 0.5-s rotation time; 1.375 pitch. Images were reconstructed using iterative reconstruction and displayed using Hybrid Viewer (Hermes Medical Solutions, Sweden) scaled to a fixed standardised uptake value (SUV) of 10 and using a standard colour table.

MTV was measured on the baseline PET scan by one observer (HI) using:

1. In-house software named ‘PET Therapy Response Assessor’ (PETTRA) developed as part of a PhD project to segment a tumour using counts with SUV ≥ 2.5 (PETTRA 2.5) as previously reported [26]
2. Commercial software ‘Hermes Hybrid 3D’ in development by Hermes Medical to segment tumours using SUV ≥2.5 (Hermes 2.5)
3. Volume with counts ≥41% of the maximum SUV within individual tumour regions (Hermes 41%) by applying a thresholding tool available within the Hermes Hybrid 3D application [33]
4. Uptake higher than the mean SUV in a 3-cm^3^ cuboid volume of interest (VOI) in the right lobe of the liver as recommended by the authors of PERCIST (Hermes PERCIST) [32]

The first three methods involved automatic segmentation of areas of tumour selected by the operator using a single-click for each region.

In the PERCIST method, the operator placed a 3-cm^3^ VOI in the right lobe of the liver. A wizard named ‘Tumour finder’ then automatically segmented all volumes within the image with uptake ≥1.5 x mean SUV + 2 standard deviations (SD) in the liver VOI. We also tested the exploratory threshold of 1 x mean SUV + 2 SD suggested [32], but found it to be too sensitive, selecting multiple areas that did not contain tumour (data not shown). If the liver showed extensive lymphoma involvement, a 1 × 1 × 2-cm VOI was placed in the descending thoracic aorta and used as the reference region instead [32].

The operator then modified volumes as required—manually removing regions that contained only physiological FDG uptake, e.g. brain or bladder, or by using editing tools to remove physiological uptake adjacent to the tumour that had been automatically included in the volume, e.g. myocardial or urinary tract and bowel uptake.

Individual tumour volumes, where more than one volume was present, were summed to calculate the total MTV. Observers were blinded to patient outcome.

### Interobserver variation

To analyse interobserver variation, a second more experienced observer (SFB) measured MTV independently from the first observer (HI) using all 3 methods available in the Hermes Hybrid 3D application in a subset of 50 patients. Five scans were randomly selected from each decile of MTV (using Hermes 2.5) to give a representative selection of high and low values. Time to complete the measurement of MTV for each method was also recorded.

### Statistical analysis

Agreement was measured between the in-house and commercial software (PETTRA 2.5 & Hermes 2.5), the three methods available in the commercial software (Hermes 2.5, Hermes 41%, Hermes PERCIST) and the different observers (HI & SFB).

The intraclass correlation coefficient (ICC) was used to measure consistency between MTV values [34]. However, since the Kolmogorov-Smirnov (KS) normality test revealed a significant non-normal distribution (*p* < 0.001), MTV values were transformed using the cube root (KS, *p* = 0.66) before calculating the ICC. Kendall's tau correlation coefficient was used to measure agreement in the ranked MTV values. Non-parametric Bland-Altman plots were used to evaluate median bias and limits of agreement (2.5% and 97.5% percentiles) from the untransformed MTV values [35].

Survival analysis was performed for all four methods of measuring MTV. PFS was defined as the time from diagnosis to the point of progression or death from any cause. OS was defined as the time from diagnosis to death from any cause. Patients still alive were censored at the date of last contact.

Receiver operating characteristic (ROC) curves were used to assess predictability of each MTV measure and identify optimal cut-offs to predict PFS. Optimal cut-off points were calculated as the minimum of the sum of squares of 1 – sensitivity and 1 – specificity (the point nearest to the top left corner of the ROC curve). Kaplan-Meier analysis was used to estimate survival time statistics (median and 5-y PFS and 5-y OS) for ‘low-’ and ‘high-MTV’ groups for each method. The log rank test was used to test if groups had significantly different survival curves. Univariate Cox regression was also applied to each MTV measure to calculate hazard ratios between the groups. *p* < 0.05 was considered to be statistically significant.

All statistics were calculated using R version 3.3.0 [36].

## Results

### Patient population

Results are available for 147 patients with a median follow up of 3.8 years (range 1.3–7.9 years). Patient clinical characteristics were as previously reported [26]. The 5-y PFS for the whole group was 65.4% and 5-y OS was 73.7%.

The values obtained for MTV using the different methods for the patient population are given in Table 1.

**Table 1:** Descriptive statistics for MTV values

Method	Mean	SD	Min.	Q1 = 25%	Median	Q2 = 75%	Max.
PETTRA 2.5	990.14	1210.24	1.50	140.53	595.12	1411.75	7357.20
HERMES 2.5	989.14	1210.27	1.08	147.17	592.48	1387.28	7348.00
HERMES PERCIST	1057.21	1599.77	0	97.75	443.61	1344.06	8365.28
HERMES 41%	255.75	340.55	0	36.69	165.76	358.25	2443.29

#### Agreement between in-house and commercial software using the same segmentation threshold (SUV ≥ 2.5)

**Fig. 1:**
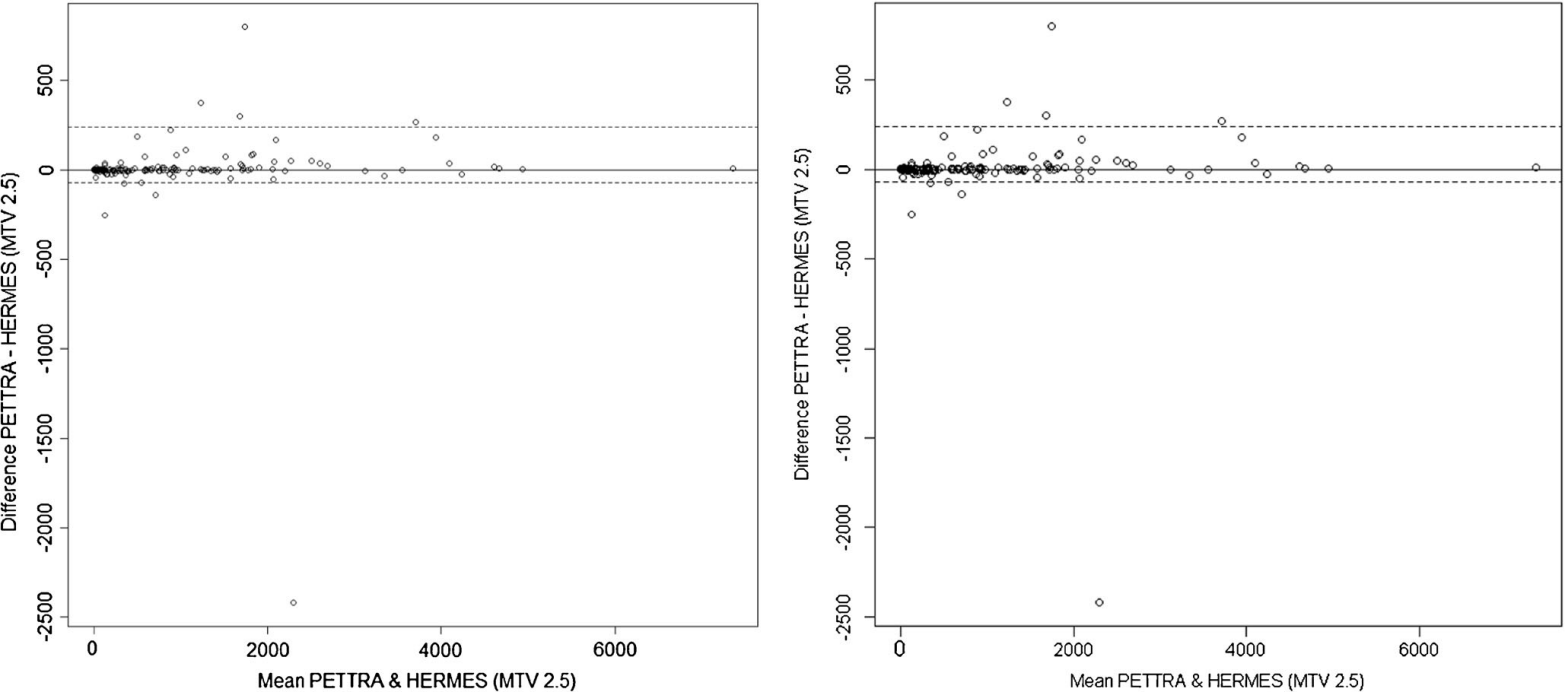
Bland-Altman plot of MTV2.5 measured using PETTRA (in-house) software and Hermes (commercial) software. The horizontal axis represents the mean of the two MTV methods and the vertical axis, the difference between them. The solid line shows the median difference (close to zero) and the dashed lines show the 95% limits of agreement (LoA). The median is very close to zero, indicating no systematic difference between the methods, and the range of LoA is relatively small compared to the scale of the MTV values, indicating a good numerical agreement in the methods among the majority of patients

There was strong agreement between the total MTV measured in our previous publication using SUV ≥ 2.5 to segment tumour with in-house software and the commercially available software (Table 2). Bland-Altman analysis (Fig. 1) showed no significant median bias nor trend in the difference in the untransformed MTV values, with a median difference of 0.03 and limits of agreement (LoA) for 2.5% and 97.5% percentiles, respectively, of −72.5 and 240.7 cm^3^.

**Table 2:** In-house (PETTRA) and commercial software (Hermes) show strong correlation and close limits of agreement (LoA) for measuring MTV using the 2.5 method. The three different methods using Hermes software also show strong correlation and LoA with one another, with the highest agreement observed between the 2.5 and PERCIST methods

	Intraclass coefficient (ICC)	Kendall’s tau	Median difference	Lower LoA	Upper LoA
PETTRA 2.5 vs. Hermes 2.5	0.99 *	0.95 *	0.03	−72.5	240.7
Hermes 2.5 vs. Hermes PERCIST	0.98 *	0.89 *	27.32	−2081.8	595.5
Hermes 2.5 vs. Hermes 41%	0.86 *	0.72 *	305.72	2.2	3770.2
Hermes PERCIST vs. Hermes 41%	0.83 *	0.73 *	246.38	−2.5	6081.3

*p < 0.001

#### Agreement between different MTV segmentation thresholds using commercial software

Agreement was strong and statistically significant between all three methods (Table 2) and strongest between the 2.5 and PERCIST methods. Rank correlation was also strongest between 2.5 and PERCIST methods with significant strong correlations between 41% and the other two methods. There was a marked difference, however, in the absolute values for MTV (Table 1) using the 41% method compared to the other methods that used either SUV ≥ 2.5 or the mean liver SUV (PERCIST). This is because the 41% method selected a smaller proportion of tumour volume, especially where there was a heterogeneous distribution. The mean and median values of the 41% method were only 26% and 28% of the values using the 2.5 method.

Nine patients categorised in the high MTV group using 2.5 were categorised as having low MTV using 41% and, conversely, 2 patients categorised as having high MTV using 41% were categorised as low MTV using 2.5. Five of these 11 patients progressed, 4 were in the high MTV group by the 2.5 method, and 1 in the high-MTV group using the 41% method.

**Fig. 2:**
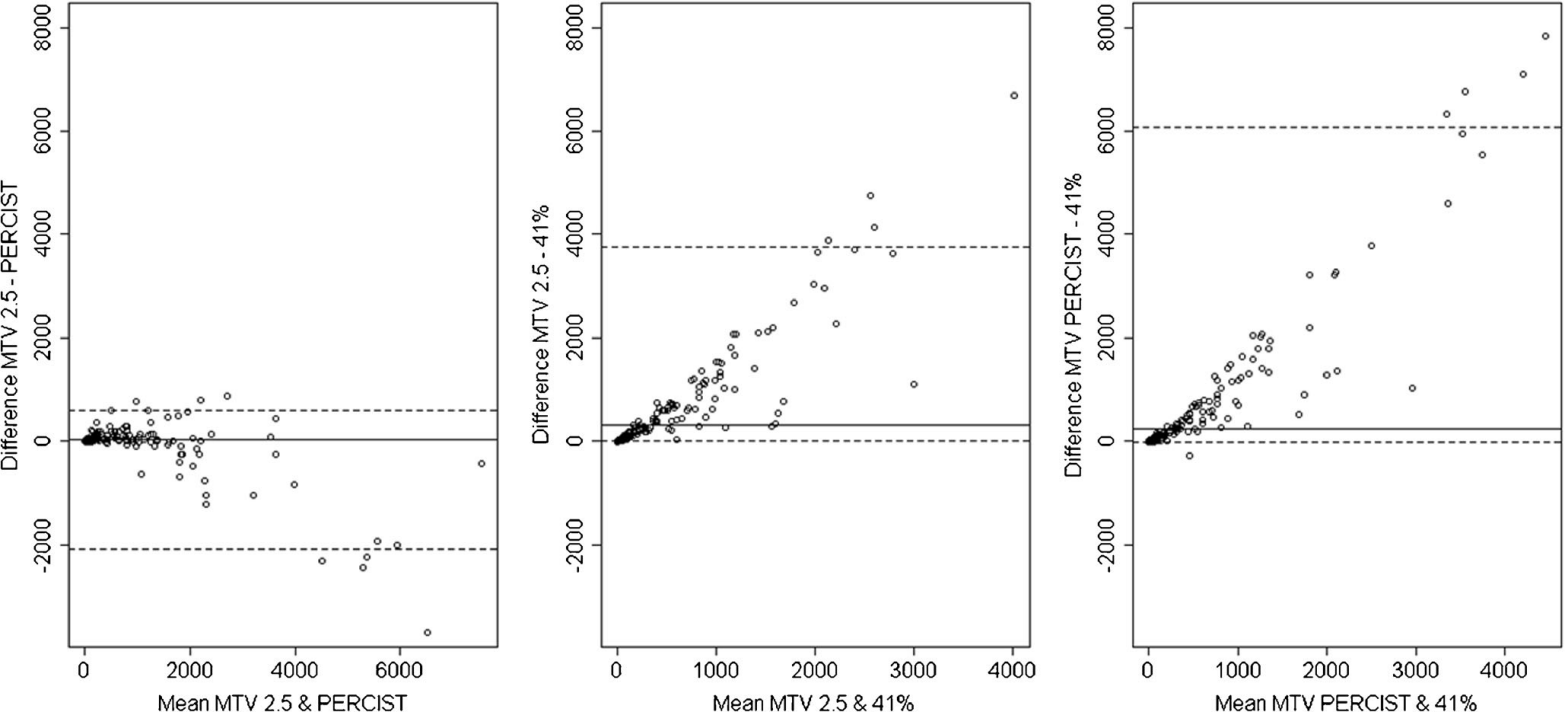
Bland-Altman plots (see Fig. 1 caption for description) comparing MTV measured by the different methods by a single observer. Compared with Fig. 1, the LoAs on each plot cover a range closer to the range of MTV values, indicating a poorer numerical match between each pair of methods. Additionally, there is an observable trend: the difference increases as the mean value increases, indicating a systematic difference dependent on the MTV

Although the SUV ≥ 2.5 and PERCIST methods showed a strong correlation, the LoAs on the Bland-Altman plot (Fig. 2a) were wide. The Bland-Altman analysis showed a clear observable trend between mean value and difference, between 41% and the other methods (Fig. 2b and c). A trend between the SUV ≥ 2.5 and the PERCIST method was also apparent (Fig. 2a). This was due to 11 patients with high disease burden, where the MTV calculated using the PERCIST method was higher than using SUV ≥ 2.5 because the liver had lower uptake in these individuals (average liver SUVmax was 1.6, average liver SUVmean was 1.0). A further 7 patients had liver involvement by lymphoma where the mediastinal blood pool, which has lower uptake than the liver, was used instead as the reference region.

#### Inter-observer variation and ease of use

There was excellent agreement between the two observers for measuring MTV with each of the methods using Hermes software. The ICCs were 0.9996, 0.9831 and 0.9984, respectively, for Hermes 2.5, Hermes 41% and Hermes PERCIST (*p* < 0.001 for all methods).

Kendall’s tau coefficients were 0.9765, 0.9027 and 0.9639, respectively (*p* < 0.001 for all methods).

**Fig. 3:**
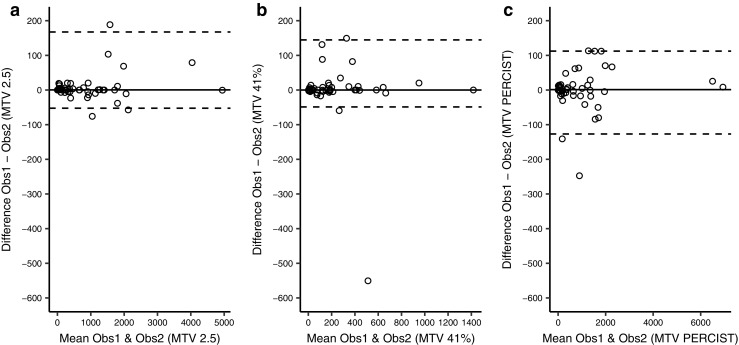
Bland-Altman plots (see Fig. 1 caption for description) comparing MTV measured by the different methods by two different observers. The median difference (solid lines) is close to zero for all three methods, indicating no systematic bias. The LoAs (dashed lines) are close, indicating good agreement

Bland-Altman plots showed a median difference of 0.4 (LoA: −52.4 to 167.5), 0.0 (LoA: –48.8 to 144.6) and 1.1 (LoA: –126.9 to 112.3) for the 3 methods, respectively (Fig. 3). No trends were observed.

The 41% method was the most time-consuming. The average time (and range) to measure total MTV using Hermes software was 2.7 (0.2–10.7) minutes for the SUV ≥ 2.5 method, 6.2 (0.4–21.6) minutes for the ≥41% method and 3.2 (0.8–8.1) minutes for the PERCIST method. The 41% method involved a two-stage process to outline the tumour with a constraining volume, find the maximum, then recontour using 41% of the maximum, rather than a single step as with the SUV ≥ 2.5 approach. It also required editing of volumes in patients where large areas of tumour involved several nodal groups with heterogenous uptake. It is recommended that where counts differ by more than 10%, regions should be subdivided to avoid underestimation of tumour volume [33]. The PERCIST method was usually the quickest overall, as it allowed automatic segmentation of regions using the wizard, except in cases where there were separate head and neck views where the observer had to delineate the regions separately on this view.

#### Prediction of prognosis - ROC & survival analysis

**Fig. 4:**
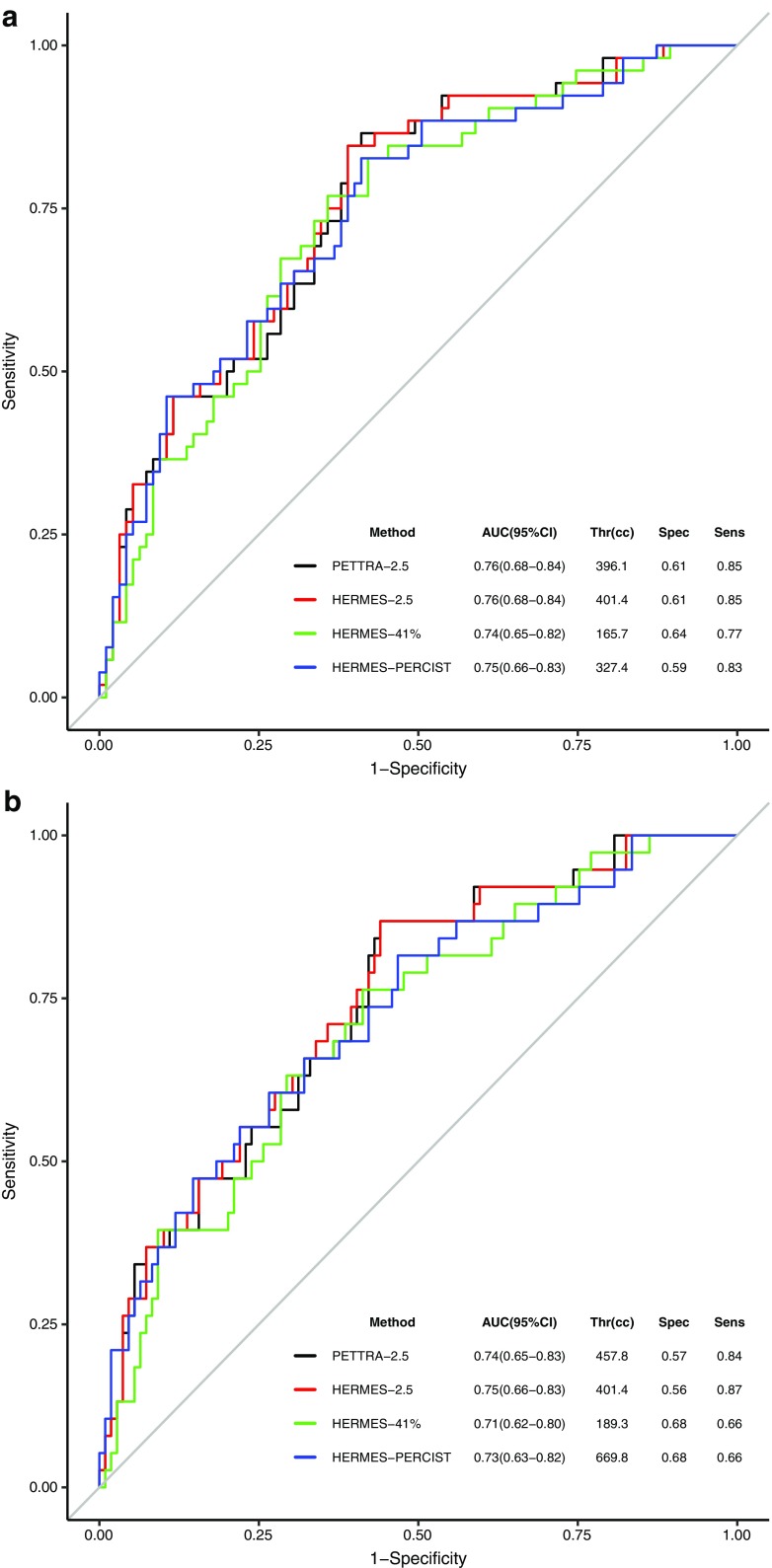
ROC curves for PETTRA 2.5, Hermes 2.5, Hermes 41% and Hermes PERCIST for a) PFS and b) OS. The tables show the area under the curve (AUC) with 95% confidence intervals (95% CI), optimum threshold value for each MTV (Thr), with associated sensitivity (sens) and specificity (spec)

The distribution and area under the ROC curves for all four methods were similar, suggesting the methods to be close in accuracy for the prediction of PFS (Fig. 4a) even though they gave different cut-offs for ‘low’ and ‘high’ MTV values. ROC curves for OS (Fig. 4b) similarly yielded almost identical curves with similar optimal thresholds for the methods, except PERCIST. The optimal threshold for PERCIST for OS (670 cm^3^) was approximately twice as high as for PFS (327 cm^3^). However, the method of choosing the optimal threshold balances both sensitivity and specificity. Considering this grouped measure and imperfect ROC curves, the PERCIST PFS threshold of 327 cm^3^ for OS was similarly optimal (specificity 53%, sensitivity 82%).

**Fig. 5:**
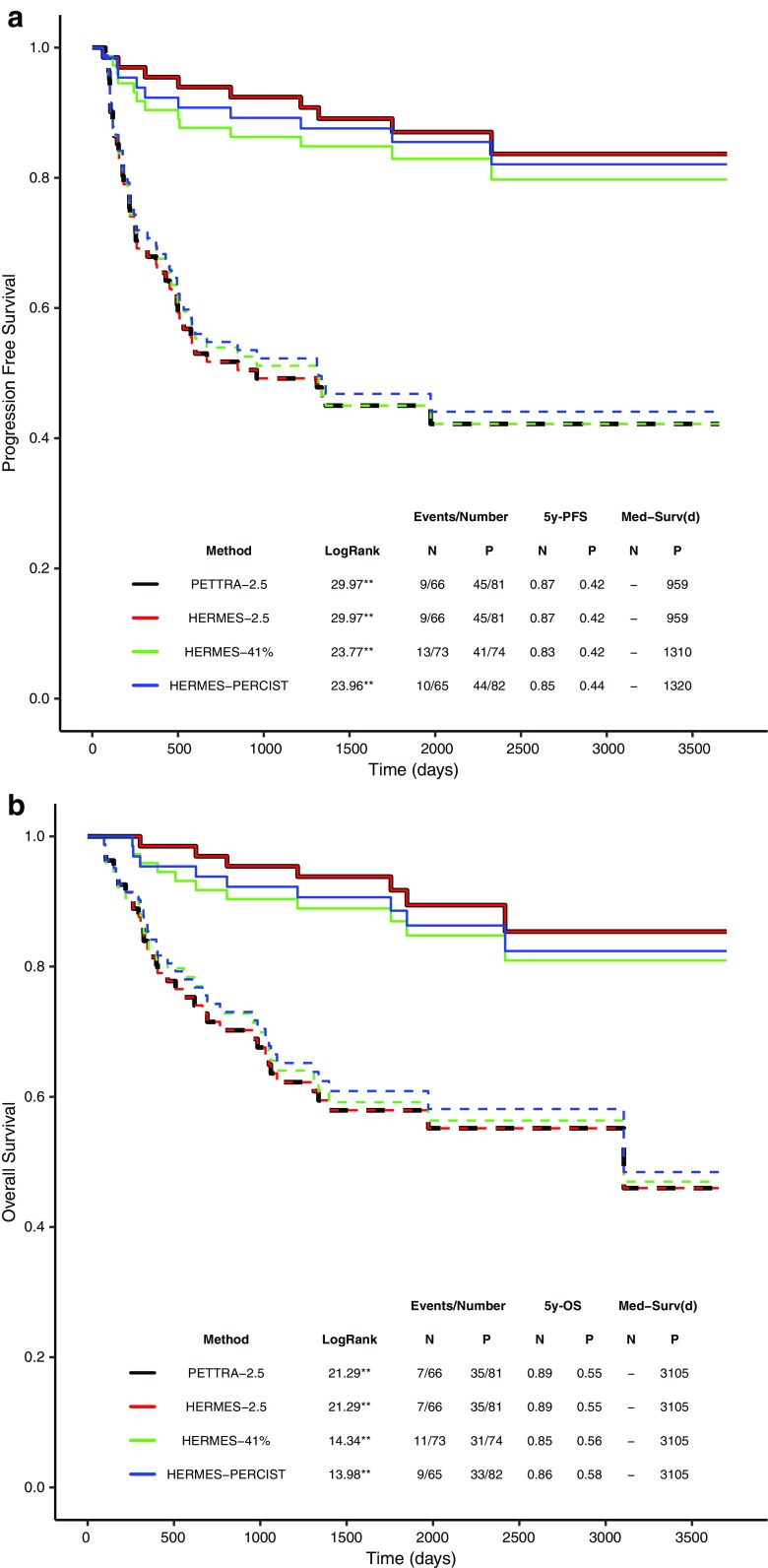
Kaplan–Meier survival curves for PETTRA 2.5, Hermes 2.5, Hermes 41% and Hermes PERCIST for a) progression-free survival (PFS) and b) overall survival (OS). Both plots use PFS-derived optimal thresholds to define high and low MTV. Solid line = low-MTV group , dotted lines = high-MTV group (defined by optimal thresholds). ** *p* < 0.001. The table shows log-rank scores from comparison of non-progressor (N) & progessor (P) for each MTV method, with number of events, 5-year PFS ([5y-PFS) and median survival in days (Med-Surv). Log-rank scores revealed significant differences in PFS between progressors & non-progressors with all methods. No non-progressor groups reached below 50% PFS (i.e. no median survival is available)

**Fig. 6:**
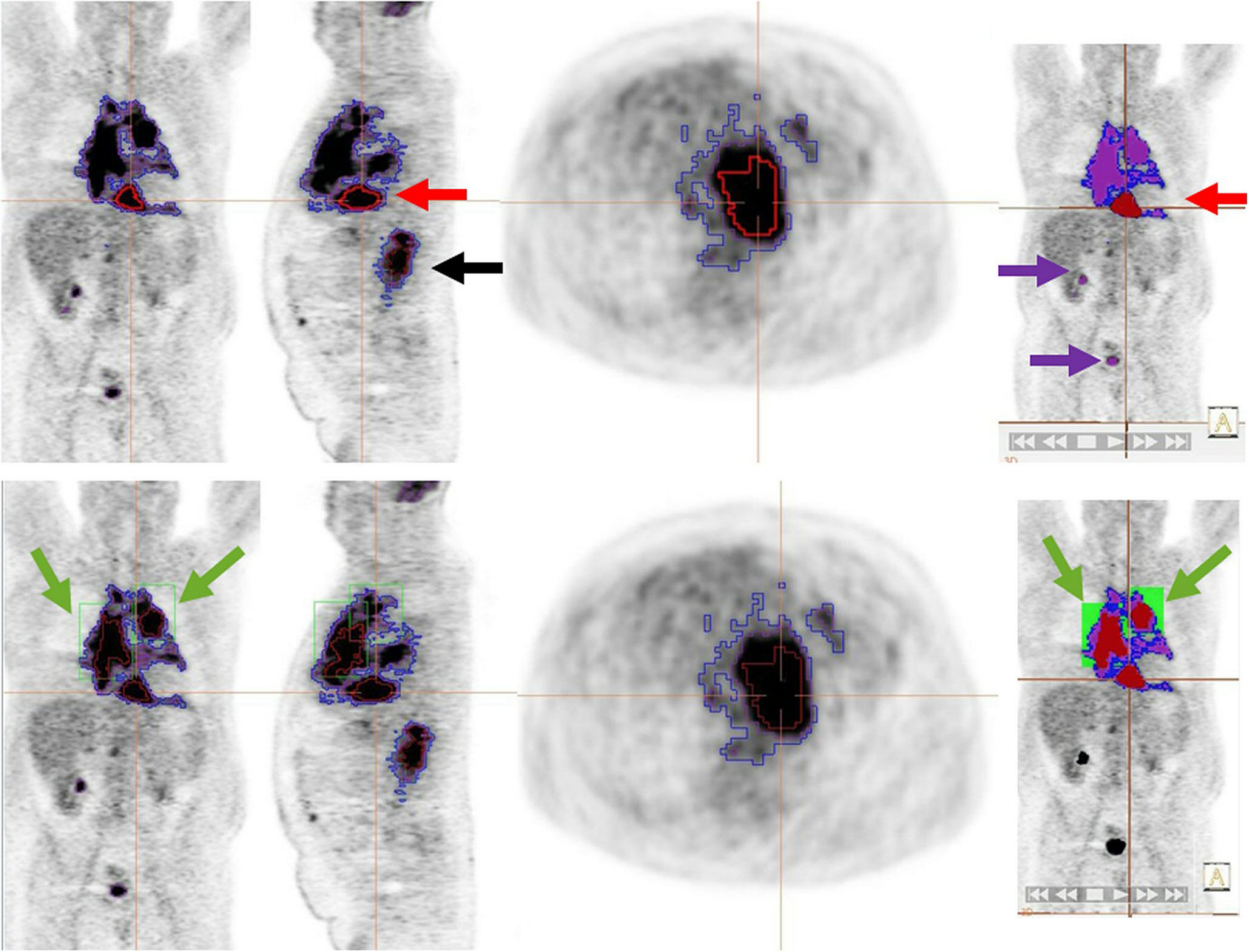
An example of a case outlined using the 2.5 method (blue), the 41% method (red) and the PERCIST method (purple) with representative coronal, sagittal, axial and 3D images. The top panel shows the initial ‘automatic’ volumes. All methods result in similar volumes for disease below the diaphragm (black arrow, sagittal view). However, for disease above the diaphragm, the MTV is grossly underestimated using the 41% method and separate bounding boxes of differing sizes have to be drawn (green boxes in the bottom panel) to delineate 2 additional volumes, increasing the time and complexity of MTV selection. The PERCIST method detects physiological uptake in the brain and urinary tract (purple arrows) which must be edited out by the observer

Kaplan–Meier analyses (Fig. 5a) showed that the patients with low MTV have a significantly longer 5y-PFS compared to the patients with high MTV, regardless of the method. The 5y-PFS was 87% versus 42% for the low- and high-MTV groups for the 2.5 method which was identical using PETTRA and Hermes software, 83% vs. 42% for the 41% method and 85% vs. 44% for the PERCIST method (Fig. 6).

Cox regression calculated the hazard ratios for PFS (high MTV compared to low MTV) to be 5.9 [2.9–12.2 95% confidence interval (CI)], 5.9 (2.9–12.2 CI), 4.8 (2.4–9.5 CI) and 4.2 (2.2–7.9 CI) for PETTRA 2.5, Hermes 2.5, Hermes PERCIST and Hermes 41% methods, respectively (all *p* < 0.001).

Patients in the low-MTV group also had significantly longer OS than patients in the high-MTV group using the optimal PFS-derived thresholds with similar separation between high- and low-MTV groups for all methods (Fig. 5b). The 5y-OS was 89% vs. 55% for the 2.5 method, 85% vs. 56% (41% method) and 86% vs. 58% (PERCIST method) (Fig. 5b).

The hazard ratios for OS were 5.5 (2.4–12.5), 5.5 (2.4–12.5), 3.7 (1.8–7.8) and 3.5 (1.8–7.0) for PETTRA 2.5, Hermes 2.5, Hermes PERCIST and Hermes 41% methods, respectively (all *p* < 0.001).

## Discussion

Baseline MTV, using FDG-PET, is a promising prognostic indicator in patients with DLBCL, which is better than using size-defined bulk [25, 26]. Tumour lesion glycolysis, which is the MTV multiplied by the mean SUV in the volume, is also prognostic [37], but appears no better than MTV in DLBCL [26, 27]. Cut-offs ranging from 220 to 600 cm^3^ have been reported to separate patients into groups with low and high baseline MTVs (Table 3) which are predictive of PFS and OS. Cut-offs have been derived using ROC curve analyses [24–27] that depend on the distribution of values in the dataset, which are influenced by patient characteristics (Table 3), with populations with worse clinical characteristics tending to have a higher optimal cut-off for MTV, but also crucially, as demonstrated in our study, on the method used to outline the tumour volume. The influence of the method of measurement on the optimal cut-off has been previously reported in 59 patients with Hodgkin lymphoma [39] and 106 patients with T cell lymphoma [40]. For clinical use, a consensus will be required on a suitable method and an optimal cut-off to define the MTV for specific lymphoma subtypes and treatment regimens, which will require validation in multicentre prospective trials.

**Table 3:** Patient clinical characteristics and methods used in studies reporting MTV in DLBCL

	N	PFS and OS of study cohort (%)	% > 60 y	% Stage III/IV	% Bulk	IPI	PS ≥ 2	Treatment	Method	Cut-off (cm^3^)	PFS by MTV (%)	OS by MTV (%)
Song 2011 [25]	169	At 3 y: PFS 74 OS 76	60	41% stage III, no stage IV or I	4% ≥ 5 cm	26% ≥ 3	25%	RCHOP	SUV ≥ 2.5	220	At 3 y: 90 vs. 56**	At 3 y: 93 vs. 58**
Sasanelli 2014 [24]	114	NA	31	82	36% ≥ 10 cm	65% ≥ 2 (aaIPI)	30%	RCHOP/RACVBP	≥ 41% SUVmax	550	At 3 y: 77 vs. 60	At 3 y: 87 vs. 60**
Song 2016 [38]	107	NA	67	100% had BMI	19%	81 ≥ 4 (NCCN-IPI)	16%	RCHOP	SUV ≥ 2.5	600	At 2 y: ~ 80 vs. 20%**	At 2 y: ~ 80 vs. 20%**
Cottereau 2016 [24]	81	At 5 y: PFS 60 OS 63	63	80	40% ≥ 10 cm	68% ≥ 2 (aaIPI)	30%	RCHOP/RACVBP	≥ 41% SUVmax	300	At 5 y: 75 vs. 42	At 5 y: 78 vs. 46**
Mikhaeel [26] and current study	147	At 5 y: PFS 65 OS 74	48	69	40% ≥ 10 cm	69% ≥ 2	30%	RCHOP	SUV ≥ 2.5	400	At 5 y: 87 vs. 42 **	At 5 y: 89 vs. 55 **

**Independent predictor in multivariate analysis for survival

BMI – bone marrow involvement, PS - performance status, RCHOP - rituximab and cyclophosphamide, doxorubicin, vincristine, prednisone, RACVBP – rituximab and doxorubicin, cyclophosphamide, vindesine, bleomycin, and prednisone

Algorithms have already been developed for segmentation of volumes for radiotherapy planning purposes in solid tumours [41, 42]. Boundaries can be chosen using an absolute SUV value or a percentage of the maximum SUV. Alternatively, more complex methods may be adopted, such as contrast-orientated, possibility theory and adaptive thresholding. No single method is likely to perform optimally in every patient, and consensus methods, such as the majority vote, have been reported to improve accuracy compared with the ‘ground truth’ of manual delineation by experts or surgical specimens [43]. In a recent publication, consensus methods performed better than the worse performing of three established automatic segmentation methods and were close to the best-performing method in all patients [43]. Five segmentations were implemented in a single software platform for evaluating patients scanned on four different cameras with lung and breast tumours and which also included eight patients with lymphoma.

So far in DLBCL, three methods have been proposed in the literature for measuring MTV [26, 32, 33]. Importantly none of these methods are vendor-specific and we have demonstrated that measurement using the 2.5 method is robust using in-house software and commercial software. Efforts are being made to develop automated freeware incorporating all the published methods [39], but whether this will be acceptable for making patient management decisions using MTV as a prognostic tool remains to be seen.

We tested these methods in a population of consecutive patients with de novo DLBCL treated with standard R-CHOP at a single institution, likely to be representative of the general patient population. We did not measure CT-based tumour burden as the CT component of the PET-CT scans were performed as low-dose non-contrast scans, in keeping with our usual clinical practice. The first method measured any activity that may be significant with a SUV greater than 2.5 [26]. The second method was derived from phantom experiments to give the best estimate of anatomical volume [33]. The third method also measured any significant activity, but using liver uptake as the threshold [32], which may be less influenced by factors that cause inaccuracy in SUV measurement, but which may be more dependent on patient preparation and metabolic status, with reduction in normal liver uptake observed when there is very high tumour burden at baseline [38].

The in-house and commercial methods for measuring MTV using the 2.5 method gave almost identical results. The PERCIST method was very close to the 2.5 method, but probably overestimated MTV in approximately 12% of patients who had low FDG uptake in the liver or liver involvement by lymphoma. The 41% method was very different in absolute MTV values compared to the other methods and was more susceptible to measurement variability when there was tumour heterogeneity.

Accordingly, we found the optimal cut-off for MTV to predict PFS ranged from 166 to 400 cm^3^. Although all three methods could predict PFS with similar accuracy in the overall study population, we found for some individual patients with very intense masses, the 41% method appeared to underestimate tumour volume compared with the other methods. The 2.5 method gave an optimal cut-off in our study population (400 cm^3^) which was in the middle of the cut-offs previously reported by Song and colleagues using this method in two publications. The first measured MTV in good-prognosis patients with no extranodal involvement (derived cut-off 220 cm^3^) [25] and the second in poor-prognosis patients, all of whom had bone marrow involvement (derived cut-off 600 cm^3^) [44]. The 41% method gave an optimal cut-off in our population which was much lower than the 550 cm^3^ [24] and 300 cm^3^ [40], respectively, reported by Meignan and colleagues in two publications. There were twice as many patients over the age of 60 in the study with the lower cut-off, which is surprising as increased age is generally associated with worse prognosis. Therefore, the cut-off might have been expected to be higher in an older population (Table 3). Other clinical characteristics were similar in these two studies. The variability in the cut-offs reported for the 41% method raise concerns that the optimal cut-off may be more dependent on how regions are selected by different groups, when there is considerable tumour heterogeneity.

There was high interobserver agreement for measuring MTV with all methods. The 41% method was the most complex to use in our experience, reflected in the time taken to measure MTV in a subset of 50 patients. The PERCIST method was usually the quickest, because it allowed automatic segmentation of all regions on the scan, using the ‘tumour finder’ wizard (©Hermes Medical Solutions). This was despite needing to edit out areas that had uptake above the liver, accounted for by areas with high physiological uptake such as the brain and bladder. Inflammatory uptake might also require editing, but we did not observe this in the patients in our study (Fig. 6).

The study confirmed that the prognostic role of baseline MTV [26] using software developed in-house could be reproduced accurately using commercially developed software. We previously found that baseline MTV was a good prognostic indicator, better than size-defined bulk [26]. Using all three methods, 5y-PFS in the current study was similar to the values reported in our earlier manuscript of 43% for patients with high MTV compared to 85% for patients with low MTV [26] and compares favourably with previous publications [24, 40]. The sensitivity and specificity of the three methods is shown in Fig. 4.

We combined baseline MTV with early response assessment at two cycles in an attempt to improve prognostic value in our previous work [26]. High MTV and failure to achieve a complete metabolic response (Deauville score 4,5) at 2 cycles was found in 31% of patients who experienced 58% of study events with 5y-PFS of only 30%. Combining MTV with baseline factors rather than early response might be a more attractive option. Recently, El-Galaly and colleagues [23] reported that combining baseline PET findings with the new National Comprehensive Cancer Network (NCCN) IPI, which splits patients according to age groups >40, >60 and >75 years and by LDH levels 1–3 or >3 times the upper limit of normal, was better at predicting prognosis than PET combined with the IPI or revised IPI. Furthermore, the number of involved extranodal sites and the presence of bone/bone marrow, pleura and female genital organ involvement was associated with inferior prognosis. Combining NCCN-IPI and MTV and possibly other baseline imaging features as suggested by el-Galaly and colleagues [23] might be even more informative.

The cell of origin is also known to influence prognosis, with the activated B cell (ABC) subtype conferring a worse prognosis than the germinal centre B cell (GCB) subtype [9]. Genetic rearrangements including overexpression of BCL2 and MYC which regulate apoptosis and proliferation area are also associated with inferior prognosis [10, 45]. Cottereau et al. reported on 81 patients [40] mostly with advanced stage DLBCL, combining molecular profiling data with MTV. High MTV using a cut-off of 300 cm^3^ (by 41% method) was associated with identical 5y-PFS of 43% in our study. The subset of 16 patients with high MTV and the ABC subtype had 5y-PFS of 30% and OS of 23%. Patients with overexpression of BCL2 and/or MYC had inferior prognosis irrespective of MTV. MTV, however, separated the 55 remaining patients into good- and intermediate-prognosis groups. This suggests a potential for the strategy of combining imaging and other biomarkers for pretreatment risk referred to as ‘Radio(gen)omics’. Evaluation will involve pooling of data to derive and validate risk estimates with international collaboration.

In summary, all the published methods for measuring MTV in DLBCL were prognostic in our study for PFS and OS. The optimal cut-off using the 2.5 method in this unselected patient population was in line with cut-offs published by another group using this method in two populations with good and poor prognosis, respectively. A limitation was that scans were acquired at 90 min, longer than currently recommended by EANM procedural guidelines [46]; nonetheless, in our hands, the 2.5 method had the advantage of being easy to use and reproducible across different software platforms and between observers. In our opinion, contouring methods based on percentages of the maximum uptake in the volume may be easier to apply in solid tumours [42, 46] than in DLBCL, where patients often present with multiple regions with heterogeneous uptake. Developments in software may overcome some of the difficulties with measurement that we encountered.

The methodology is evolving and will require prospective validation in sufficiently large patient cohorts combined with other prognostic factors, to determine whether robust pre-treatment risk estimates can be identified to select patients in whom to test alternative treatments including novel agents.
